# A novel acetyl xylan esterase enabling complete deacetylation of substituted xylans

**DOI:** 10.1186/s13068-018-1074-3

**Published:** 2018-03-22

**Authors:** Fakhria M. Razeq, Edita Jurak, Peter J. Stogios, Ruoyu Yan, Maija Tenkanen, Mirjam A. Kabel, Weijun Wang, Emma R. Master

**Affiliations:** 1 Department of Chemical Engineering and Applied Chemistry, University of Toronto, 200 College Street, Toronto, ON M5S 3E5 Canada; 2 Department of Bioproducts and Biosystems, Aalto University, Kemistintie 1, 00076 Aalto Espoo, Finland; 3 Department of Food and Environmental Sciences, University of Helsinki, P.O. Box 66, 00014 Helsinki, Finland; 4 Laboratory of Food Chemistry, Wageningen University, Bornse Weilanden 9, 6708 WG Wageningen, The Netherlands

**Keywords:** Acetyl xylan esterase, α-Glucuronidase, Glucuronic acid, Polysaccharide utilization loci, Xylan, SGNH hydrolase

## Abstract

**Background:**

Acetylated 4-*O*-(methyl)glucuronoxylan (GX) is the main hemicellulose in deciduous hardwood, and comprises a β-(1→4)-linked xylopyranosyl (Xyl*p*) backbone substituted by both acetyl groups and α-(1→2)-linked 4-*O*-methylglucopyranosyluronic acid (MeGlc*p*A). Whereas enzymes that target singly acetylated Xyl*p* or doubly 2,3-*O*-acetyl-Xyl*p* have been well characterized, those targeting (2-*O*-MeGlc*p*A)3-*O*-acetyl-Xyl*p* structures in glucuronoxylan have remained elusive.

**Results:**

An unclassified carbohydrate esterase (FjoAcXE) was identified as a protein of unknown function from a polysaccharide utilization locus (PUL) otherwise comprising carbohydrate-active enzyme families known to target xylan. FjoAcXE was shown to efficiently release acetyl groups from internal (2-*O*-MeGlc*p*A)3-*O*-acetyl-Xyl*p* structures, an activity that has been sought after but lacking in known carbohydrate esterases. FjoAcXE action boosted the activity of α-glucuronidases from families GH67 and GH115 by five and nine times, respectively. Moreover, FjoAcXE activity was not only restricted to GX, but also deacetylated (3-*O*-Ara*f*)2-*O*-acetyl-Xyl*p* of feruloylated xylooligomers, confirming the broad substrate range of this new carbohydrate esterase.

**Conclusion:**

This study reports the discovery and characterization of the novel carbohydrate esterase, FjoAcXE. In addition to cleaving singly acetylated Xyl*p*, and doubly 2,3-*O*-acetyl-Xyl*p*, FjoAcXE efficiently cleaves internal 3-*O*-acetyl-Xyl*p* linkages in (2-*O*-MeGlc*p*A)3-*O*-acetyl-Xyl*p* residues along with densely substituted and branched xylooligomers; activities that until now were missing from the arsenal of enzymes required for xylan conversion.

**Electronic supplementary material:**

The online version of this article (10.1186/s13068-018-1074-3) contains supplementary material, which is available to authorized users.

## Background

**Fig. 1:**
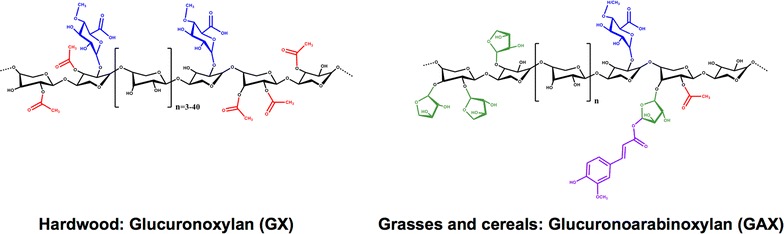
Schematic structures of major xylan types. The xylosyl backbone is shown in black and ramifications with acetyl groups (red), 4-*O*-methylglucopyranosyluronic acid (MeGlcpA; blue) and arabinosyl units (green) linked with ferulic acid groups (purple) are indicated

Hemicelluloses represent the second main polysaccharide component in lignocellulosic biomass after cellulose. In addition to a source of carbohydrates for fuels and chemicals, reported applications of hemicelluloses range from rheology modifiers and packaging films, to hydrogels as well as nutrient additives in food and feed [1, 2]. Despite their broad application potential, hemicelluloses remain relatively underutilized, in part due to their diverse and heterogeneous compositions [3]. 4-*O*-Methyl-glucuronoxylan (GX) is the main hemicellulose in deciduous trees, and contains β-(1→4)-linked xylopyranosyl (Xyl*p*) backbone units that can be acetylated and/or substituted by α-(1→2)-linked 4-*O*-methylglucopyranosyluronic acid (MeGlc*p*A). Although species variations occur, approximately one of every ten Xyl*p* units are substituted with MeGlc*p*A, and six of every ten Xyl*p* are acetylated at the *O*-2 and/or *O*-3 positions [4–7] (Fig. 1). By contrast, glucuronoarabinoxylans (GAX) dominate in cereal grains such as corn, and are characterized by Xyl*p* backbone residues that are decorated with l-arabinofuranosyl substituents at either the *O*-3 or both *O*-2 and *O*-3 positions. To a lesser extent, these xylans can be substituted with 5-*O*-*trans*-feruloyl-l-arabinofuranose and/or an oligomeric side chain at the *O*-3 position [8], as well as Glc*p*A and MeGlc*p*A residues and acetyl groups at the *O*-2, *O*-3 or both positions of Xyl*p* subunits [9–11] (Fig. 1). The presence of side groups along the xylan backbone impact several properties of corresponding biopolymers, including water solubility, rheology, adsorption behavior, nutrient value, and enzymatic conversion to monosaccharides [12].

Enzymatic conversion of xylans to monosaccharides, oligosaccharides, or defined polymeric structures requires the concerted action of carbohydrate-active enzymes (CAZymes; http://www.cazy.org) from multiple CAZyme families [13]. CAZymes that target side groups of GX and GAX include the following: α-l-arabinofuranosidases from glycoside hydrolase (GH) families GH3, GH43, GH51, GH54, and GH62, α-glucuronidases from the families GH67 and GH115, and acetyl xylan esterases (AcXEs) that have been primary reported in carbohydrate esterase (CE) families CE1, CE4–CE6, and CE16 [14–18]. Whereas α-glucuronidases from family GH115 target the α-(1→2)-linkage between MeGlc*p*A and mono-substituted Xyl*p* at internal and end positions of GX, GH67 activity is restricted to such linkages at the non-reducing end of xylan and/or corresponding oligosaccharides [7]. Similarly, CEs acting on GX have been grouped according to the positions they target (e.g., acting on *O*-2 and *O*-3 monoacetylated Xyl*p*, versus targeting 2,3-di-*O*-acetylated Xyl*p*) [19]. Certain family CE16 enzymes also show activity towards non-reducing (2-*O*-MeGlc*p*A)3-*O*-acetyl-Xyl*p* positions [19–22]; however, in these cases, acetyl group migration from *O*-3 to *O*-4 on the non-reducing Xyl*p* could not be ruled out [5, 19, 23]. Critically, to date, there are no known esterases that efficiently target internal (2-*O*-MeGlc*p*A)3-*O*-acetyl-Xyl*p* structures, nor α-glucuronidases that release MeGlc*p*A from acetylated Xyl*p*, which hinders selective release of MeGlc*p*A substituents for material applications and full conversion of GX to fermentable monosaccharides [7, 19, 24]. Similarly, CEs capable of targeting acetyl groups adjacent to arabinosyl substituents and oligomeric structures in GAX remain to be uncovered.

Polysaccharide utilization loci (PULs) comprise a physically linked set of bacterial genes that encode CAZymes and other proteins that work in concert to modify and degrade specific polysaccharides and/or oligosaccharides [25]. Predicted PULs were introduced to the CAZyme database in 2015 (http://www.cazy.org/PULDB/) [26], and have emerged as especially rich regions within genome and metagenome sequences for enzyme discovery. For example, within the last year alone, novel activities towards pectin [27], xylan [28], galactomannan [29], chitin [30], and β-glucans [31] were discovered.

Herein, we mined PUL sequences with the specific aim to finally uncover carbohydrate esterases that efficiently target (2-*O*-MeGlc*p*A)3-*O*-acetyl-Xyl*p* positions internal to GX. Briefly, we identified PULs listed in the PULDB that encode CAZyme families known to target GX (i.e., GH10, GH115), and recombinantly expressed those sequences marked as having unknown function along with a predicted signal sequence for secretion. Ultimately, FjoAcXE encoded by *Flavobacterium johnsoniae* was selected and shown to cleave acetyl groups of internal and terminal (2-*O*-MeGlc*p*A)3-*O*-acetyl-Xyl*p* structures of GX, leading to synergistic impacts on measured activities for both family GH67 and GH115 α-glucuronidases. The discovery of FjoAcXE thus completes the arsenal of CAZymes required for GX conversion to defined structures or full hydrolysis to monosaccharides. Moreover, the deacetylation activity of FjoAcXE on xylooligomers from corn fiber confirmed its ability to target acetylated Xyl*p* structures also substituted by an oligomeric side chain, demonstrating the relevance of this unclassified CE activity for the conversion of diverse, complex xylans.

## Methods

### Materials

*para*-Nitrophenol (*p*NP) alkyl esters were purchased from the following sources: *p*-nitrophenyl acetate (C_2_) (Sigma, St. Louis, MO, USA; N8130), *p*-nitrophenyl butyrate (C_4_) (Sigma, N9876), *p*-nitrophenyl hexanoate (C_6_) (TCI-EP, Tokyo, Japan; H0484), *p*-nitrophenyl octanoate (C_8_) (Sigma, 21742), *p*-nitrophenyl decanoate (C_10_) (Sigma, N0252), *p*-nitrophenyl dodecanoate (C_12_) (Sigma, 61716), *p*-nitrophenyl myristate (C_14_) (Sigma, 70124), and *p*-nitrophenyl palmitate (C_16_) (Sigma, N2752). In addition, 4-methylumbelliferyl acetate (4-MUA) was purchased from Sigma (M0883) and 4-methylumbelliferone (4-MU) was purchased from Aldrich (M1381).

Acetylated monosaccharide substrates were purchased from the following sources: β-d-glucose pentaacetate (Aldrich, 285943), β-d-xylopyranose tetraacetate (Synthose Inc., Toronto, ON, Canada; TX534) and 1,2,3,4-tetra-*O*-acetyl-l-rhamnopyranose (Synthose Inc., TH212). Acetylated glucurono-xylooligosaccharides (Ac-XOS) were isolated by steam extraction of milled chips from *Eucalyptus* wood [32], or mixed hardwood kindly provided by Prof. Bradley Saville (University of Toronto, Canada) [33]. Isolation and characterization of xylooligosaccharides from corn fibers were previously described [8].

The family GH115 α-glucuronidase from *Amphibacillus xylanus* (AxyAgu115) was purified as previously described [34]. The family CE6 acetylxylan esterase from *Orpinomyces* sp. (E-AXEAO) was purchased from Megazyme (Bray, Ireland); the family GH67 α-glucuronidase from *Cellvibrio japonicus* (PRO-E0069; CjGlcA67A) was purchased from PROZOMIX Ltd (Haltwhistle, UK).

### Candidate selection

The PUL database (PULDB, http://www.cazy.org/PULDB) [26] was searched for PULs that contained at least two of the predicted xylan-active CAZyme families of interest, such as GH10, GH43, GH67, and GH115. From the obtained list of PULs, all proteins of unknown function were extracted and analyzed using Signal P4.1 [35]; those containing a predicted signal sequence for secretion were further analyzed based on the presence of Pfam domains, sequence length, and the type of CAZymes present on the corresponding PUL. Based on these criteria, FjoAcXE (PULDB ID: Fjoh_3879; GenBank ID: ABQ06890.1) was selected for recombinant protein expression. A structural model of FjoAcXE was built using PHYRE2.0 [36] for the CBM-like domain, using PDB 2O14 as template, and Modeller 9.19 [37] for the CE/catalytic domain using PDB 2O14 and PDB 1K7C [38]. The model was displayed with PyMOLv1.7.4.5 Edu (PyMOL Molecular Graphics System, Schrödinger, LLC).

### Gene synthesis and molecular cloning

The gene encoding FjoAcXE lacking the predicted signal sequence (residues 1–21) was codon optimized for expression in *Escherichia coli* K12 using IDT Codon Optimization Tool (http://www.idtdna.com/CodonOpt). Fifteen base pair extensions homologous to the p15TV-L vector (GenBank ID: EF456736.1) (T7, 5′-TTGTATTTCCAGGGC and T7term, 5′-CAAGCTTCGTCATCA) were added to each end of the sequence and the gene was synthesized as gBlock^®^ gene fragments (Integrated DNA Technologies, Inc., Coralville, IA, USA). gBlock fragments were cloned into p15TV-L using the In-Fusion^®^ HD EcoDry™ Cloning Kit (Clontech Laboratories, Inc., Palo Alto, CA, USA). The resulting plasmid was transformed into *E. coli* HST08 Stellar™ Competent Cells (Clontech Laboratories, Inc.), and the sequence was verified using DNA sequencing service at the Center of Applied Genomics at the SickKids Hospital (Toronto, ON, Canada).

### Protein expression and purification

*E. coli* BL21(λDE3) codon plus strain harboring p15TV-L-FjoAcXE was propagated at 37 °C in 8 L of Luria–Bertani (LB) Broth–Miller (BioShop) supplemented with 33 µg/mL chloramphenicol and 100 µg/mL ampicillin until the OD_600_ reached 0.6–0.8. Cultures were cooled on ice for 5 min; additional 33 µg/mL chloramphenicol and 100 µg/mL ampicillin were added, and recombinant expression was induced overnight at 16 °C with 0.5 mM isopropyl β-d-1-thiogalactopyranoside. Cells were harvested by centrifugation at 8967×*g* (Beckman Coulter, JLA-8.1000) for 15 min at 4 °C and the pellet (approx. 25 g fresh weight) was frozen at − 80 °C. The pellet was then suspended in binding buffer (50 mM HEPES pH 7.5, 300 mM NaCl, 5% glycerol and 5 mM imidazole) and the cells were disrupted by sonication (100 amplitude, 5 s ON and 5 s OFF for 20 min). Cell debris was removed by centrifugation at 27,167×*g* (Beckman Coulter, JA-25.5 rotor) for 15 min at 4 °C and supernatant was filtered through Acrodisc^®^ Syringe Filters with 0.45-µm Supor^®^ membrane (Pall Corporation).

The sample was loaded onto 5-mL HisTrap HP (GE Healthcare) pre-equilibrated with binding buffer. A combination of step-wise and gradient elutions using elution buffer (50 mM HEPES pH 7.5, 300 mM NaCl, 5% glycerol and 300 mM imidazole) was performed on Biologic DuoFlow™ chromatography system (BioRad) where the column was washed with 4 column volumes (CV) of 100% binding buffer, and then increasingly substituted with elution buffer as follows: 5% elution buffer for 4 CV, 10% elution buffer for 6 CV and then gradient elution from 10 to 100% elution buffer over 12 CV. All steps were done at 1 mL/min, protein elution was monitored at A_280 nm_ and 2 mL fractions were collected throughout the entire run. The resulting fractions were analyzed by 12% SDS-PAGE; selected fractions were pooled and then exchanged to 25 mM HEPES (pH 8.0) using 10 kDA Jumbosep™ centrifugal devices (Pall Corporation). The sample was then further purified using a 1.3-mL UNO™ Q ion exchange column (BioRad) pre-equilibrated with 25 mM HEPES pH 8.0 (Buffer A). Following a wash using 11.5 CV of Buffer A, a step-wise gradient elution was performed at 1 mL/min on a Biologic DuoFlow™ using 25 mM HEPES pH 8.0 with 1 M NaCl as elution buffer (Buffer B), where 0–50% of Buffer B was passed over 15.4 CV, and then up to 100% Buffer B over 6 CV. The samples were collected as 1-mL fractions throughout the entire run and purity was checked with 12% SDS-PAGE. The purified sample was exchanged to 10 mM HEPES pH 7.5 containing 300 mM NaCl before being flash frozen in liquid nitrogen and stored at − 80 °C.

Protein concentration was measured using the Bradford assay with bovine serum albumin as a standard [39]. The identity of purified FjoAcXE was then confirmed by peptide mass fingerprinting using an easy-nLC-1000 (ThermoFisher Scientific, Bremen, Germany) equipped with a 10.5-cm PicoTip Emitter Silica Tip packed in-house with C18 media coupled online to a Q-Exactive mass spectrometer (ThermoFisher Scientific) [40].

### Enzyme activity measurements using *p*NP aliphatic fatty acid esters and 4-MUA

200 mM stock solutions for *p*NP alkyl esters from C2 to C12 were prepared in 100% DMSO, whereas *p*NP substrates from C14 to C16 were prepared in 1:1 (v/v) of isopropanol:acetonitrile. Reactions were performed in 50 mM HEPES (pH 7.0) with 2 mM substrate and were initiated by adding 0.5 µg of FjoAcXE; the final reaction volume was 200 µL. Reactions were conducted at pH 7.0 to minimize non-enzymatic de-esterification of *p*NP substrates over the prolonged incubation (i.e., 2 h). Reactions continued for 2 h at 30 °C, and absorbance was measured continuously at 410 nm. Reaction mixtures without FjoAcXE were used as a blank.

Kinetic parameters of FjoAcXE were determined using *p*NP-acetate and 4-MUA. For *p*NP-acetate, reactions contained approximately 0.06 µg (1.32 × 10^−6^ µmol) of FjoAcXE in 50 mM HEPES (pH 8.0) and were initiated by adding 0.05 mM to 10 mM *p*NP-acetate. The final reaction volume was 200 µL, and absorbance at 410 nm was read continuously for 40 min at 30 °C.

For 4-MUA, reactions contained approximately 0.02 µg (4.41×10^−7^ µmol) of FjoAcXE in 50 mM HEPES (pH 8.0) and final of 10% DMSO. The enzyme dose was chosen to ensure a linear relationship between reaction time and product release [41]; reactions were then initiated by adding 0.01–1.5 mM of 4-MUA. The final reaction volume was 200 µL, and absorbance at 354 nm was read continuously for 40 min at 30 °C. Kinetic parameters were calculated using Michaelis–Menten equation and Graphpad Prism 5 software (La Jolla, CA, USA).

### Optimum reaction conditions

The pH optimum of FjoAcXE was tested using 100 mM Tris, 50 mM MES, 50 mM acetic acid, and 50 mM sodium acetate trihydrate buffer with pH range of 3.5–9.5. Reactions containing 0.5 mM 4-MUA were initiated by adding 5 µg of FjoAcXE, and continued for 20 min at 30 °C; the final reaction volume was 200 µL. 4-MUA was selected to determine the pH optimum of FjoAcXE given the relative stability of this substrate under alkaline conditions [41]. For all reactions using 4-MUA, absorbance was measured at 354 nm and the reaction mixture without FjoAcXE was used as a blank.

To measure pH stability, solutions containing 4 µg of FjoAcXE were incubated for 16 h at 4 °C in 100 mM Tris, 50 mM MES, 50 mM acetic acid, and 50 mM sodium acetate trihydrate buffer adjusted to pH 3.5–9.5; the final solution volume was 40 µL. After incubation, residual enzyme activity was tested in reactions containing 0.5 mM 4-MUAc in 50 mM HEPES (pH 8.0). Reactions were initiated by adding 1 µg of FjoAcXE from each treatment and continued for 20 min at 30 °C; the final reaction volume was 200 µL.

The temperature stability of FjoAcXE was tested by suspending 4 µg of FjoAcXE in 50 mM HEPES (pH 8.0) and incubating each suspension (40 µL final volume) for up to 16 h at 20, 30, or 40 °C, or up to 60 min at 50, 60, or 70 °C. After the incubation, residual activity was tested in reactions containing 0.5 mM 4-MUAc in 50 mM HEPES (pH 8.0). Similar to pH stability, reactions were initiated by adding 1 µg of FjoAcXE from each treatment, and continued for 20 min at 30 °C; the final reaction volume was 200 µL.

### Effect of divalent ions, detergents, and organic solvents

The metal-free apoenzyme of FjoAcXE was prepared as described in Wang et al. [42]. Briefly, 0.05 g of CHELEX 100 (Sigma) was added to 1 mg of FjoAcXE and incubated at room temperature for 20 min. The effect of metal ions was tested in reactions containing 0.5 mM 4-MUA in 50 mM HEPES (pH 8.0). Reactions were initiated by adding 5 µg of FjoAcXE, and continued for 20 min at 30 °C in the presence of 1 mM of the following metal ions: Ag^2+^, Ca^2+^, Cd^2+^, Co^2+^, Cu^2+^, Fe^3+^, Mg^2+^, Mn^2+^, Ni^2+^, and Zn^2+^ (all as chloride salts, except for Ag^2+^, which was a nitrate). The final reaction volume was 200 µL, and control samples included CHELEX treated and untreated FjoAcXE with and without 20 mM EDTA. Absorbance was measured at 354 nm and the reaction mixture without FjoAcXE was used as a blank.

The chemical stability of FjoAcXE was tested in reactions containing 0.5 mM 4-MUAc in 50 mM HEPES (pH 8.0). Reactions were initiated by adding 0.5 µg of FjoAcXE, and continued for 20 min at 30 °C in the presence of 30% (v/v) ethanol, 30% (v/v) isopropanol, 1% (w/v) SDS, 1% (w/v) dithiothreitol (DTT), 1% (v/v) Tween-20, 1% (v/v) Tween-80, and 1% (v/v) TritonX-100; the final reaction volume was 200 µL.

### Activity on acetylated monosaccharides

Reactions comprised 50 mM HEPES (pH 8.0) and 0.1% (w/v) of β-d-glucose pentaacetate (final concentration 2.5 mM), β-d-xylopyranose tetraacetate (final concentration 3.14 mM), or 1,2,3,4-tetra-*O*-acetyl-l-rhamnopyranose (final concentration 3 mM). Reactions were initiated by adding 0.5 µg of FjoAcXE; the final reaction volume was 30 µL. Reactions continued for 20 min at 30 °C, and were stopped by boiling for 10 min. The samples were quickly centrifuged and release of acetic acid was then measured in the supernatant using the Acetic Acid kit (K-ACETRM, Megazyme). Reaction mixtures without FjoAcXE were used as a blank for each substrate.

### ^1^H-NMR analysis of FjoAcXE activity on acetylated (glucurono)-xylooligosaccharides

^1^H-NMR was performed to monitor the activity of FjoAcXE and AxyAgu115A on Ac-XOS substrates. Reactions comprised 1% (w/v) acetylated (glucurono)-xylooligosaccharides in 50 mM HEPES buffer (pH 7.0) and 10 µg of each protein; the final reaction volume was 400 µL. The reaction continued for 20 h at 30 °C and gentle shaking. Reaction mixtures without enzyme were used as negative controls. Following the incubation, samples were filtered through Acrodisc^®^ syringe filters with 0.2-µm Supor^®^ membrane (Pall Corporation), and lyophilized. The samples were then dissolved in 300 µL D_2_O and transferred into 3-mm NMR tubes (Norell) for analysis using an Agilent DD2 700 MHz spectrometer equipped with a triple resonance HCN cold probe with a scan number of 64, relaxation delay of 1 s and acquisition time of 4.5 s. The data were obtained using VnmrJ 4.0 (Agilent) and analyzed with MestReNova 10.0 (Mestrelab Research). The HDO peak at 4.790 was used as internal standard. The change in signal intensity in the regions between 5.4 and 4.4 ppm corresponding to acetylated Xyl*p* residues in the anomeric region of the spectrum, and 2.30–2.05 ppm region corresponding to the acetyl group methyl protons were used to assign proton chemical shifts, as reported in Uhliariková et al. [24].

### Impact of FjoAcXE on α-glucuronidase activity towards (glucurono)-xylooligosaccharides

FjoAcXE, CjGlcA67A, AxyAgu115A, and E-AXEAO were tested alone and as pairs of carbohydrate esterase and α-glucuronidase activities. Reactions comprised 1% (w/v) Ac-XOS in 50 mM HEPES (pH 7.0), which is within one pH unit from the optimum pH of each enzyme. Reactions were initiated by adding 0.5 µg of each enzyme; the final reaction volume was 30 µL. Reactions continued for 20 min at 30 °C and were stopped by boiling for 10 min. The samples were quickly centrifuged and release of acetic acid and d-glucuronic acid were measured using Acetic Acid (K-ACETRM, Megazyme) and d-glucuronic acid/d-galacturonic acid (K-URONIC, Megazyme) assay kits, respectively. Reaction mixtures without enzymes were used as blanks.

### Matrix assisted laser desorption time of flight mass spectrometry (MALDI-TOF) analysis of FjoAcXE activity on feruloylated xylooligosaccharides

Reactions comprised 50 mM HEPES (pH 8.0), 1% (w/v) of feruloylated xylooligomers from corn fiber, and were initiated by adding 1 µg of FjoAcXE; the final reaction volume was 50 µL. Reactions continued for 20 min at 30 °C with gentle shaking, and were stopped by boiling the samples at 100 °C for 10 min. Reaction mixtures without enzyme were used as negative controls. One microliter of each reaction sample (after desalting with AG 1-X8 anion exchange resin; Bio-Rad, Hercules, CA, USA) was then mixed with 2 µL matrix solution [2,5-dihydroxybenzoic acid, 10 mg/mL H_2_O/ACN (3:7)] on a stainless-steel metal plate and allowed to dry under a constant stream of air. MALDI-TOF was performed using an Ultra-flex3 instrument (Bruker Daltonics, Bremen, Germany) equipped with a nitrogen laser of 337 nm and operated in the positive mode. The system was controlled by FlexAnalysis software. Calibration was performed with a mixture of maltodextrins 480–3000 Da (Elicityl, Crolles, France). After a delayed extraction time of 130 ns, positive ions were accelerated with 22 kV voltage and detected using reflector mode.

## Results and discussion

### Candidate selection and recombinant protein production

**Fig. 2:**
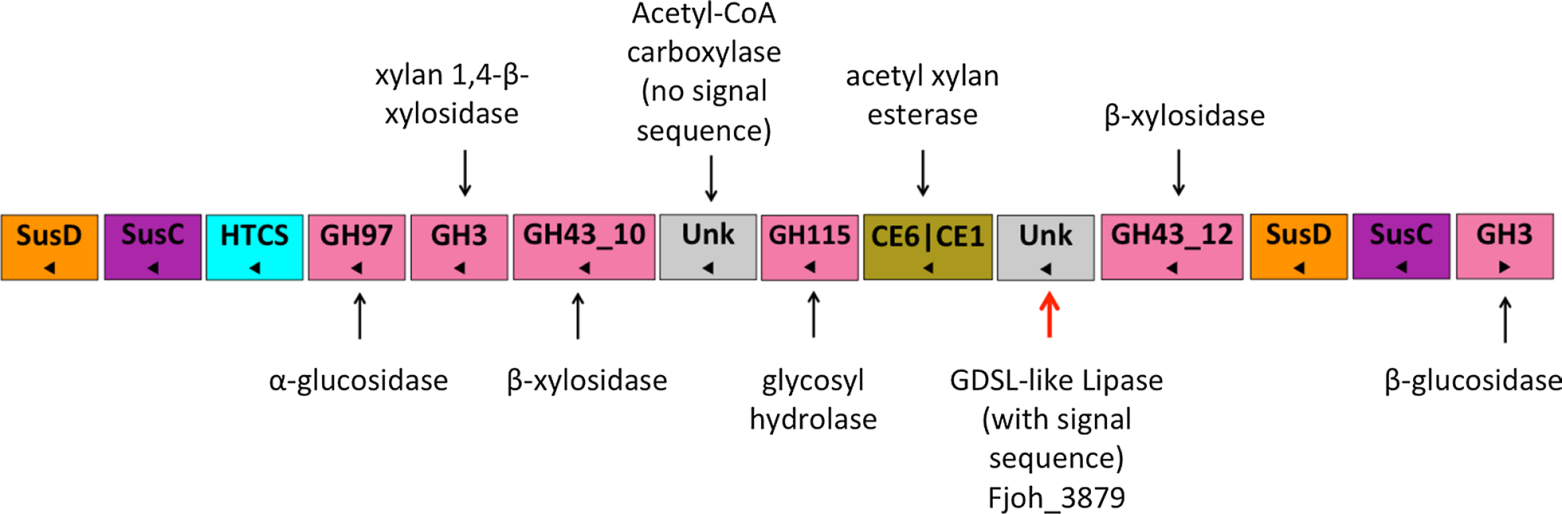
PUL20 *Flavobacterium johnsoniae* showing possible functions of predicted carbohydrate-active enzymes (CAZymes). Proteins annotated as having unknown function (unk) are shown in gray. The red arrow indicates the gene encoding FjoAcXE (Fjoh_3879). Predicted CAZymes include glycoside hydrolase (GH) and carbohydrate esterase (CE) families that comprise xylan-active enzymes. A vertical slash between two modules represents multidomain CAZymes, and arrows indicate whether the gene is found on the sense (right pointing triangle) or antisense (left pointing triangle) strand

**Fig. 3:**
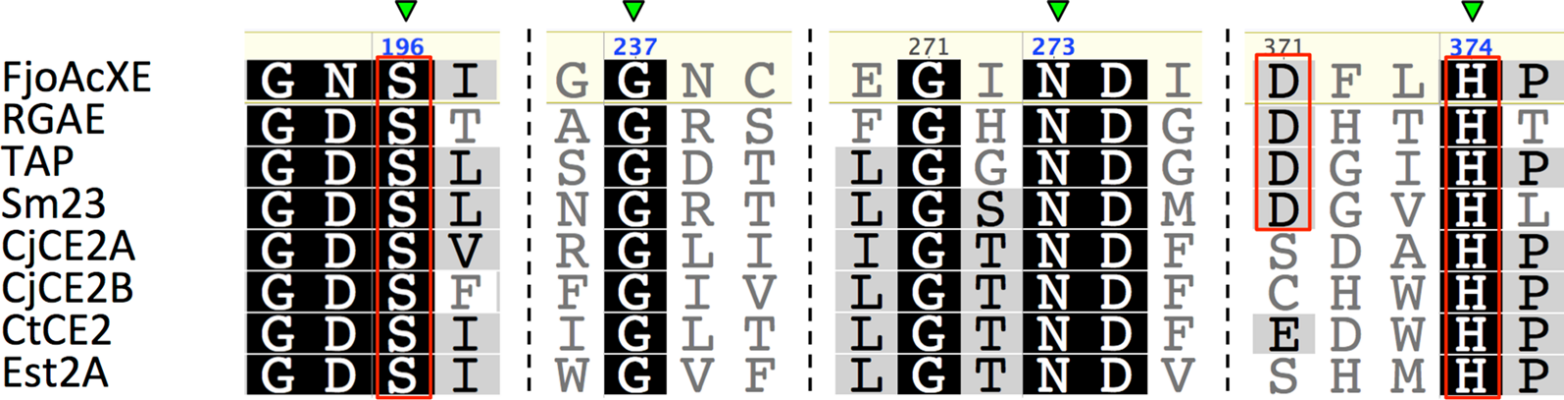
Alignment of FjoAcXE with structurally and biochemically characterized SGNH hydrolase-type esterases. RGAE: rhamnogalacturonan acetylesterase from *Aspergillus aculeatus* (PDB code 1DEO) [52]; TAP: Thioesterase I/Protease I/Lysophospholipase L1 from *E. coli* (PDB code 1IVN) [53, 54]; Sm23: arylesterase from *Sinorhizobium meliloti* (PDB code 4TX1) [55]; CjCE2A and CjCE2B: acetylxylan esterase from *Cellvibrio japonicus* (PDB codes 2WAA and2W9X, respectively) [43]; CtCE2: acetylxylan esterase from *Ruminiclostridium thermocellum* (PDB code 2WAO) [43]; Est2A: acetylxylan esterase from *Butyrivibrio proteoclasticus* (PDB code 3U37) [56]. Conserved SGNH residues (green triangle) and catalytic Ser-His dyad or Ser-Asp-His triad (red squares) are highlighted. Sequences were aligned using MAFFT v.7.017 [57]

FjoAcXE was marked as a protein of unknown function within PUL20 from *Flavobacterium johnsoniae* UW 101 on PULDB, also comprising CAZyme families GH43, GH115, CE1 and CE6 (Fig. 2). In addition to a predicted signal sequence for secretion (residues 1–21), the FjoAcXE sequence contained a predicted SGNH hydrolase-type esterase domain belonging to GDSL-like lipase/acylhydrolase family (Pfam domain PF13472) (Fig. 3). FjoAcXE shares less than 30% sequence identity to CE families listed in the CAZyme database and has not been assigned to a CAZyme family. Using Phyre2.0, however, FjoAcXE was predicted to adopt a structure most similar to CEs from families CE2 and CE12 (between residues 188–393, the C-terminus) as well as a CBM-like structure at the N-terminus (residues 59–180). Guided by the Phyre2.0 result, we generated a family of models of the FjoAcXE catalytic domain using CE12 enzyme structures as templates and the Modeller program; one such model is shown in Additional file 1: Fig. S1. The models recapitulated the α/β/α-fold characteristic of SGNH hydrolases and the position of the Ser-His-Asp triad; however, they varied in the conformation of three loops surrounding the catalytic site: residues 201–208, 238–241, and 368–373. These loops are expected to shape the size of the catalytic pocket and play a role in substrate preference. In particular, the 238–241 region is located between the catalytic Ser196 and Asn273; the latter residue is thought to participate with Gly237 and the amide of Ser196 in forming an oxyanion hole to stabilize the *O*-acetylserine intermediate in the catalytic mechanism of CE enzymes [43]. As well, the 201–208 loop is located near the CBM-like domain and may play a role in substrate positioning in cooperation with that domain. The model places the catalytic site to face the CBM-like domain, indicating that this N-terminal region could play a role in placement of substrates appropriately for catalysis (Additional file 1: Fig. S1). Notably, however, affinity gel electrophoresis (AGE) using a wide range of polysaccharides did not reveal carbohydrate binding by FjoAcXE (Additional file 2: Fig. S2). Nevertheless, the SGNH family displays a wide range of hydrolytic functions [44], and given our search for CE activities targeting resistant (2-*O*-MeGlc*p*A)3-*O*-acetyl-Xyl*p* structures in GX, FjoAcXE was selected for recombinant production and biochemical characterization.

### General properties of FjoAcXE

Recombinant FjoAcXE was functionally expressed in *E. coli* with an N-terminal His_6_-tag and purified to homogeneity with approximate yield of 6 mg/g of fresh cell pellet. The predicted molecular mass of recombinant FjoAcXE is 45.2 kDa, which was consistent with that estimated by SDS-PAGE (Additional file 3: Fig. S3). Whereas no or negligible activity was measured on tested polysaccharides and *p*NP-glycosides (Additional file 4: Fig. S4, Additional file 5: Fig. S5, respectively), clear activity was observed on *p*NP-acetate followed by *p*NP-butyrate (Additional file 6: Fig. S6); notably, lack of activity on longer chain alkyl esters is consistent with esterase rather than lipase activity [45].

Given the similar activity of FjoAcXE on *p*NP-acetate and 4-MUA (Table 1), and the relative stability of 4-MUA compared to *p*NP-acetate [41], 4-MUA was used to evaluate the effect of pH, temperature, metal ions, and chemical reagents on activity of FjoAcXE.

**Table 1:** Kinetic parameters of FjoAcXE on 4-MUA and *p*NP-acetate

Substrate	Specific activity (µmol/min/mg)	*k*_cat_ (s^−1^)	*K*_m_ (mM)	*k*_cat_/*K*_m_ (s^−1^ mM^−1^)
4-MUA	126.3 ± 5.9	95.1 ± 4.4	0.8 ± 0.1	114.7
*p*NP-acetate	160.7 ± 4.0	120.8 ± 3.0	0.9 ± 0.1	127.9

*n* = 3; errors represent standard deviation

**Fig. 4:**
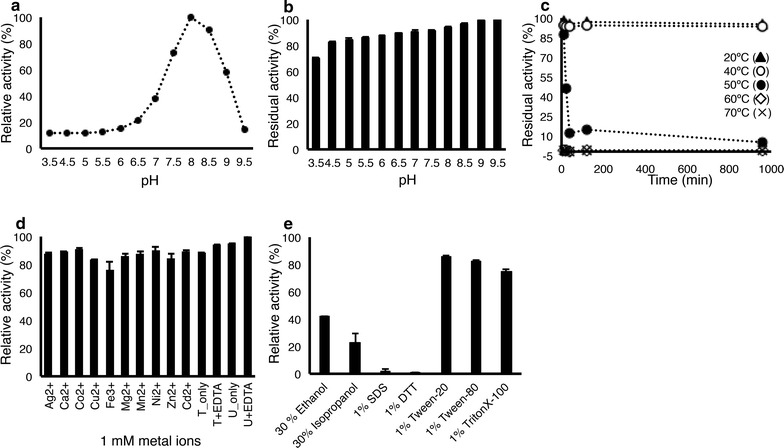
General biochemical properties of FjoAcXE. For optimum pH (**a**) and pH stability (**b**), the samples were tested using 100 mM Tris, 50 mM MES, 50 mM acetic acid, and 50 mM sodium acetate trihydrate universal buffer, with pH range of 3.5–9.5. Temperature stability (**c**), effect of metal ions (**d**), and effect of selected solvents and reactants (**e**), were measured using 0.5 mM 4-MUA in 50 mM HEPES (pH 8.0) where reactions proceeded for 20 min at 30 °C. Absorbance at 354 nm was measured and the reaction mixture without protein was used as a blank. *n* = 3; error bars correspond to standard deviation

Whereas FjoAcXE activity was highest at pH 8.0 (Fig. 4a), FjoAcXE retained over 80% of its activity after 16 h of pre-incubation at pH 4.5 to pH 9.5 (Fig. 4b). Furthermore, FjoAcXE retained 100% of its initial activity after 16 h at 20–40 °C; only 50% activity was retained after 10 min at 50 °C and no residual activity was detected after 5 min at 60 °C (Fig. 4c). The biochemical properties of FjoAcXE were similar to previously reported SGHN hydrolases [46–49] and consistent with the soil and freshwater occurrence of the source organism (*Flavobacterium johnsoniae*). Most notably, FjoAcXE shows significant pH stability (Fig. 4b) compared to other reported SGNH hydrolases, such as Est19 from *Bacillus* sp., which shows substantial loss of activity after 1-h pre-incubation at pH below 6.0 and above 10.0 [50]. None of the metal ions tested significantly affected FjoAcXE activity (Fig. 4d) and addition of 1% Tween-20, Tween-80, and Triton-X-100 resulted in only 30% loss of activity of FjoAcXE (Fig. 4e). The overall pH and surfactant stability of FjoAcXE may offer advantages in applications ranging from prebiotics, detergents, as well as biofuels [51].

### Activity of FjoAcXE on acetylated mono- and oligosaccharides

**Fig. 5:**
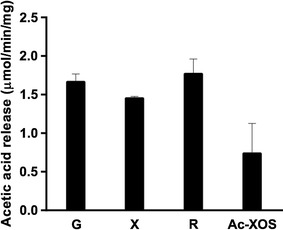
Activity of FjoAcXE on acetylated monosaccharides and xylooligosaccharides. Reactions (30 µL) comprised 0.5 µg of FjoAcXE in 50 mM HEPES (pH 8.0) and 0.1% (w/v) of β-d-glucose pentaacetate (G), β-d-xylopyranose tetraacetate (X), 1,2,3,4-tetra-*O*-acetyl-l-rhamnopyranose (R), or acetylated xylooligosaccharides from Eucalyptus (Ac-XOS); reactions were incubated at 30 °C for 20 min. Release of acetic acid was measured using acetic acid kit (K-ACETRM; Megazyme). *n* = 3; error bars correspond to standard deviation

Rates of FjoAcXE action towards fully acetylated monosaccharides and partially acetylated xylooligosaccharides were also comparable (Fig. 5), indicating that FjoAcXE has low susceptibility to steric hindrance. Moreover, activity of FjoAcXE on acetylated xylooligosaccharides supports its designation as an acetyl xylan esterase.

**Fig. 6:**
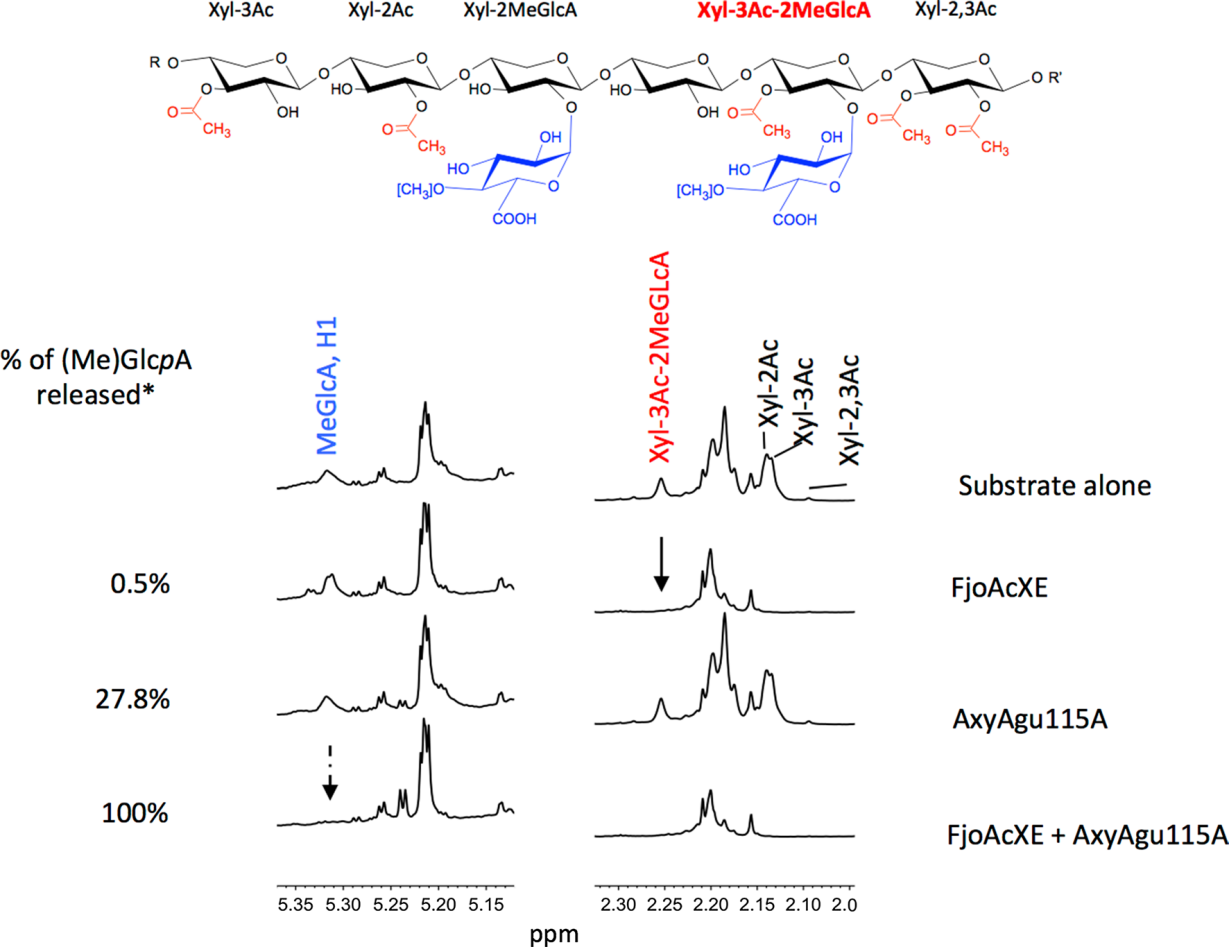
^1^H-NMR spectrum of 1% (w/v) hot water extracted glucuronoxylan following treatment with FjoAcXE. The solid arrow indicates the unique ability of FjoAcXE to deacetylate 3-*O*-acetylated Xyl*p* 2-*O*-substituted with MeGlc*p*A. The dashed arrow shows synergistic effect of FjoAcXE with AxyAgu115A [34]. Reactions were performed in 50 mM HEPES (pH 7.0) for 20 h at 30 °C; reactions were then lyophilized and suspended in 0.3 mL HDO. *100% represents 0.29 mg/mL release of (Me)Glc*p*A. Peak assignments were made as described previously [19, 21, 24]

To then evaluate the regioselectivity of FjoAcXE action, acetyl group release from specific positions within oligosaccharides of acetylated glucuronoxylan was monitored by ^1^H-NMR. Remarkably, in addition to targeting 2-*O*-acetyl-Xyl*p*, 3-*O*-acetyl-Xyl*p*, and 2,3-*O*-acetyl Xyl*p* positions, FjoAcXE fully released the acetyl group from (2-*O*-MeGlc*p*A)3-*O*-acetyl-Xyl*p* structures (Fig. 6). Such complete activity towards (2-*O*-MeGlc*p*A)3-*O*-acetyl-Xyl*p* structures has not been previously reported, and shows that FjoAcXE was not impacted by the steric hindrance presented by MeGlc*p*A that has plagued AcXE enzymes characterized to date.

**Fig. 7:**
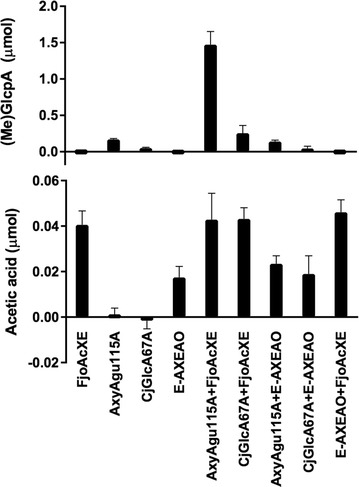
Measurement of glucuronic acid and acetic acid released from 1% acetylated xylooligosaccharides from Eucalyptus (Ac-XOS). Reactions (30 µL) contained 0.5 µg of each protein in 50 mM HEPES (pH 7.0) and were incubated for 20 min at 30 °C. Glucuronic acid released was measured using the d-glucuronic acid/d-galacturonic acid (K-URONIC, Megazyme) and acetic acid released was measured using the acetic acid kit (K-ACETRM, Megazyme). *n* = 3; error bars correspond to standard deviation

Action of FjoAcXE towards (2-*O*-MeGlc*p*A)3-*O*-acetyl-Xyl*p* structures was further explored through synergistic action of FjoAcXE with AxyAgu115A and CjGlcA67A. The commercial CE6, E-AXEAO, which was previously shown to deacetylate glucuronoxylan at all positions except (2-*O*-MeGlc*p*A)3-*O*-acetyl-Xyl*p* structures, was used for comparison [22]. MeGlc*p*A release by AxyAgu115A from oligosaccharides of acetylated glucuronoxylan (Ac-XOS) increased nearly nine times when in the presence of FjoAcXE (Fig. 7). Similarly, MeGlc*p*A release from Ac-XOS by CjGlcA67A increased nearly five times in the presence of FjoAcXE. The higher impact of FjoAcXE activity on MeGlc*p*A release by AxyAgu115A compared to CjGlcA67A is consistent with the ability of FjoAcXE to target both internal and terminal (2-*O*-MeGlc*p*A)3-*O*-acetyl-Xyl*p* structures. By contrast, addition of E-AXEAO did not impact AxyAgu115A or CjGlcA67A activity (Fig. 7), consistent with the lack of E-AXEAO activity towards (2-*O*-MeGlc*p*A)3-*O*-acetyl-Xyl*p* structures.

### MALDI-TOF analysis of FjoAcXE towards xylooligomers from corn fiber

**Fig. 8:**
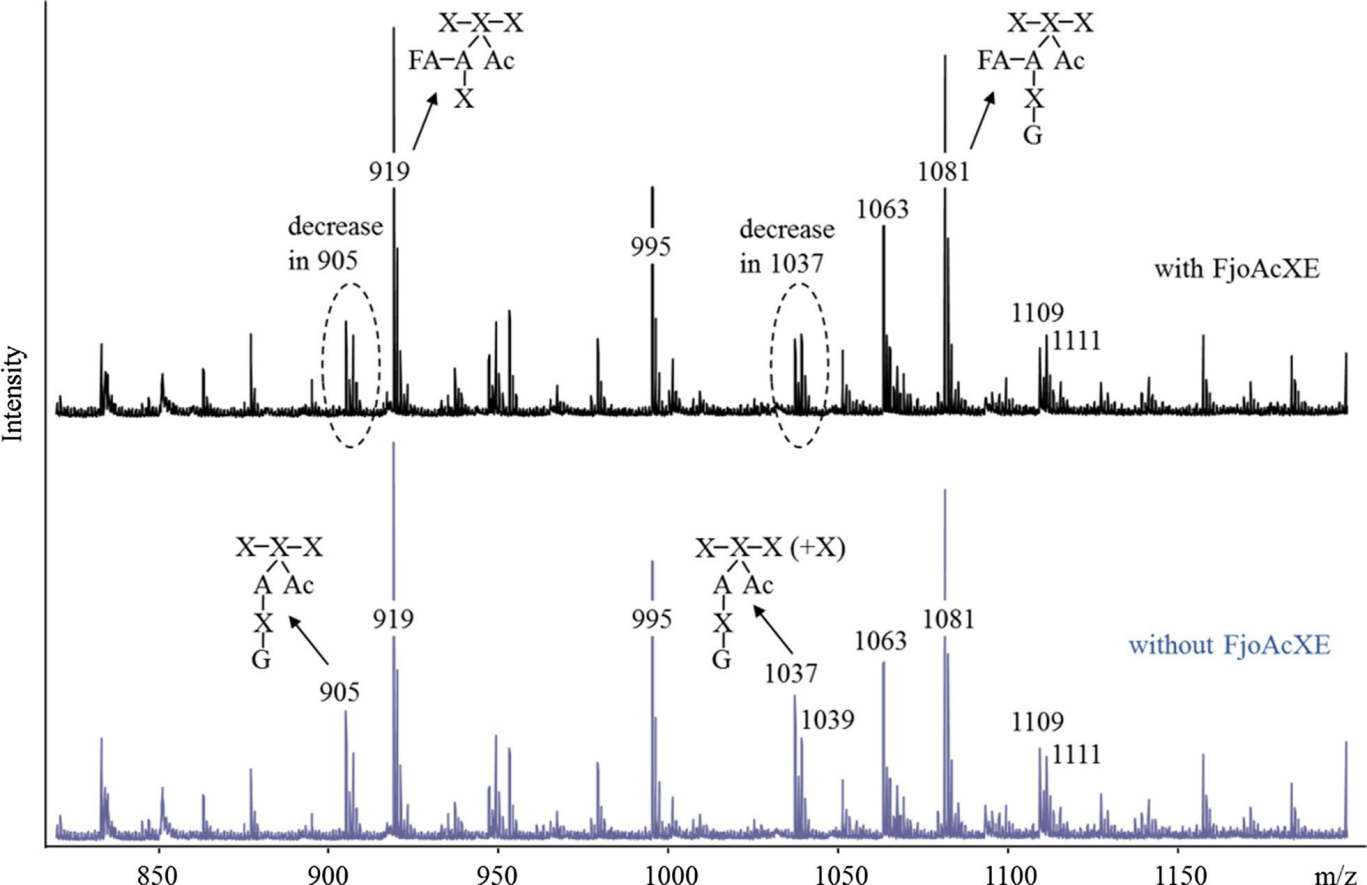
MALDI-TOF spectra of feruloylated and/or acetylated AX oligomers before (**a**) and after incubation with FjoAcXE (**b**). Acetylated structures are shown based on Appeldoorn et al. [8]; *P* pentose, *A* arabinose, *G* galactose, *Ac* acetyl, *FA* feruloyl. The *m/z* values represent sodium adducts

Given the efficient release of typically recalcitrant (2-*O*-MeGlc*p*A)3-*O*-acetyl-Xyl*p* structures, FjoAcXE activity was also tested using complex oligosaccharides recovered from feruloyated arabinoxylans, which were previously recovered and described in detail [8]. Based on the earlier, detailed characterization of the corresponding feruloyated xylooligomers by both NMR and ESI–MS, the exact structures transformed by FjoAcXE could be identified herein by MALDI-TOF [8]. In particular, a decrease in acetylated xylo-arabino-oligomers corresponding to peak (*m/z*) values 905 and 1037 was identified after treatment with FjoAcXE (Fig. 8). These results indicate that FjoAcXE is also able to target 2-*O*-acetyl-Xyl*p* structures where the same Xyl*p* is substituted at the *O*-3 position by arabinosyl residues; however, release of the acetyl group that neighbors the feruloylated side group was not detected [8] (Fig. 8).

## Conclusions

An unclassified protein from a polysaccharide utilization locus predicted to promote xylan deconstruction was biochemically characterized and shown to harbor novel carbohydrate esterase activity, which was missing from the arsenal of enzymes required for GX conversion. In addition to promoting α-glucuronidase activity through release of acetyl groups from internal (2-*O*-MeGlc*p*A)3-*O*-acetyl-Xyl*p* structures, FjoAcXE also targeted (3-*O*-Ara*f*)2-*O*-acetyl-Xyl*p* of feruloylated xylooligomers. The novel ability of FjoAcXE to release acetyl groups of Xyl*p* backbone residues that are further substituted by either MeGlc*p*A or a neutral oligomeric side chain could be explained by the loop regions predicted to surround the catalytic site of the enzyme and to play a role in substrate preference. On-going efforts to solve the FjoAcXE structure, and to characterize predicted homologs of FjoAcXE, will help confirm these predictions and likely uncover additional protein features that determine the broad substrate range of this new carbohydrate esterase activity.

## Additional files

**Additional file 1: Fig. S1.** Structural model of FjoAcXE. Top view shows a cartoon only view, button shows CE domain as solvent-exposed surface representation. The CE domain is shown in darker shade, CBM-like domain in lighter shade. The catalytic triad and SGNH motif residues are shown in red, with expected hydrogen bonds between catalytic triad S196-D371-H374 shown as dashes. Three loops impinging on the active site cleft are coloured black and labeled. The model was built with Modeller for the CE domain and Phyre2 for the CBM.

**Additional file 2: Fig. S2.** Affinity gel electrophoresis. 5 µg of FjoAcXE was run for 2 h at 90 V on 7.5% (w/v) native polyacrylamide gel (25 mM Tris, 250 mM glycine buffer (pH 8.3) containing 0.01% of each substrate; gels were then stained with Coomassie Blue G. Bovine serum albumin (BSA) was used as a reference. MGX = 4-*O*-(methyl)-glucuronoxylan; OSX = oat spelt xylan; CMC = carboxymethylcellulose, WAX = wheat arabinoxylan.

**Additional file 3: Fig. S3.** Purified FjoAcXE is approximately 45.2 kDA.

**Additional file 4: Fig. S4.** FjoAcXE activity screen against 0.5% (w/v) of selected polysaccharides. Reactions (50 µL) contained 5 µg of FjoAcXE, 50 mM HEPES (pH 8.0), and 0.5% w/v of each substrate, and were incubated for 16 h at 30 °C. Reducing sugars were measured using 1% final PAHBAH reagent [58]. BEX = beechwood xylan (Sigma, X4252); OSX = oat spelt xylan (Sigma, X0627); CMC = carboxymethylcellulose (Megazyme, P-CMC4 M); β-glucan (low viscosity; from barley; Megazyme, P-BGBL); starch (from corn; Sigma-Aldrich, S4126); pectin (from apple; Sigma, 76282); WAX = wheat arabinoxylan (high viscosity; Megazyme, P-WAXYH); arabinan (from sugarbeet; Megazyme, P-ARAB); glucomannan (low viscosity; from konjac; Megazyme, P-GLCML); galactomannan (from guar, GD28; Megazyme, enzyme modified); xyloglucan (amyloid, from tamarind seed; Megazyme, P-XYGLN); arabinogalactan (acacia gum, Sigma, G9752).

**Additional file 5: Fig. S5.** FjoAcXE activity screen against selected *p*NP substrates. Reactions (200 µL) contained 5 µg of FjoAcXE, 50 mM HEPES (pH 8.0), and 2 mM of each substrate. Absorbance was measured after 2 h at 30 °C.

**Additional file 6: Fig. S6.** Screen of FjoAcXEA activity towards selected *p*NP alkyl esters showing activity on short chain (< C4) substrates consistent with esterase rather than lipase activity. Reactions (200 µL) contained 0.5 µg of FjoAcXE, 50 mM HEPES (pH 8.0), and 2 mM of each substrate. Absorbance at 410 nm was measured after 2 h at 30 °C. *p*NP acetate (C_2_), *p*NP butyrate (C_4_), *p*NP hexanoate (C_6_), *p*NP octanoate (C_8_), *p*NP decanoate (C_10_), *p*NP dodecanoate (C_12_), *p*NP myristate (C_14_), and *p*NP palmitate (C_16_). *n* = 3; error bars correspond to standard deviation.
