# Supplementary figures and images for: A novel acetyl xylan esterase enabling complete deacetylation of substituted xylans
Source: Biotechnol Biofuels. 2018 Mar 22;11:74. doi: 10.1186/s13068-018-1074-3 (PMC5863359; doi:10.1186/s13068-018-1074-3)

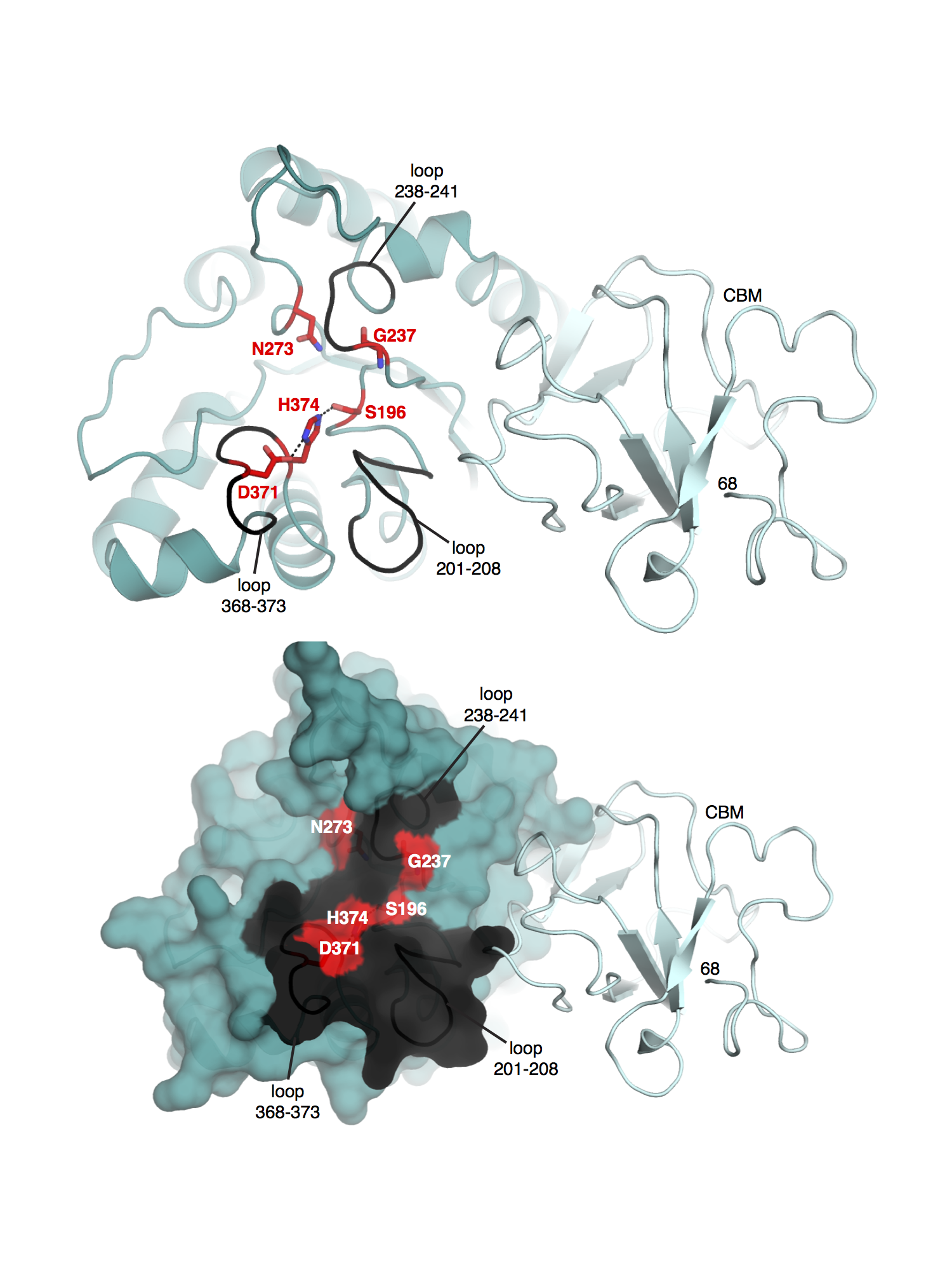

Supplement: Supplementary file 1 — Additional file 1: Fig. S1. Structural model of FjoAcXE. Top view shows a cartoon only view, button shows CE domain as solvent-exposed surface representation. The CE domain is shown in darker shade, CBM-like domain in lighter shade. The catalytic triad and SGNH motif residues are shown in red, with expected hydrogen bonds between catalytic triad S196-D371-H374 shown as dashes. Three loops impinging on the active site cleft are coloured black and labeled. The model was built with Modeller for the CE domain and Phyre2 for the CBM. [file 13068_2018_1074_MOESM1_ESM.tiff]

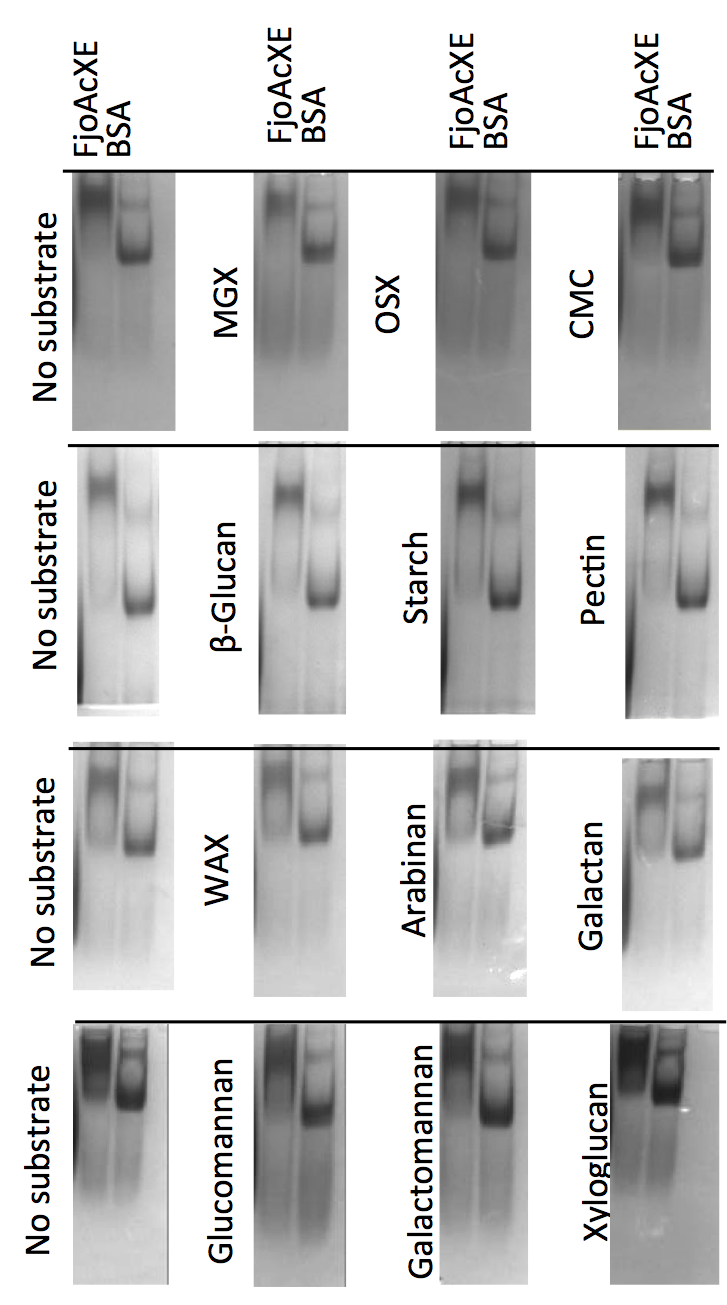

Supplement: Supplementary file 2 — Additional file 2: Fig. S2. Affinity gel electrophoresis. 5 µg of FjoAcXE was run for 2 h at 90 V on 7.5% (w/v) native polyacrylamide gel (25 mM Tris, 250 mM glycine buffer (pH 8.3) containing 0.01% of each substrate; gels were then stained with Coomassie Blue G. Bovine serum albumin (BSA) was used as a reference. MGX = 4-O-(methyl)-glucuronoxylan; OSX = oat spelt xylan; CMC = carboxymethylcellulose, WAX = wheat arabinoxylan. [file 13068_2018_1074_MOESM2_ESM.tiff]

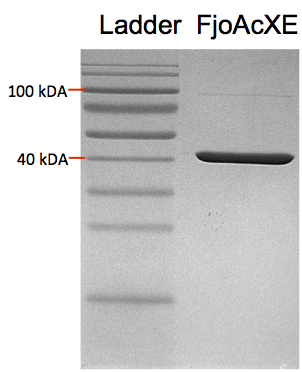

Supplement: Supplementary file 3 — Additional file 3: Fig. S3. Purified FjoAcXE is approximately 45.2 kDA. [file 13068_2018_1074_MOESM3_ESM.tiff]

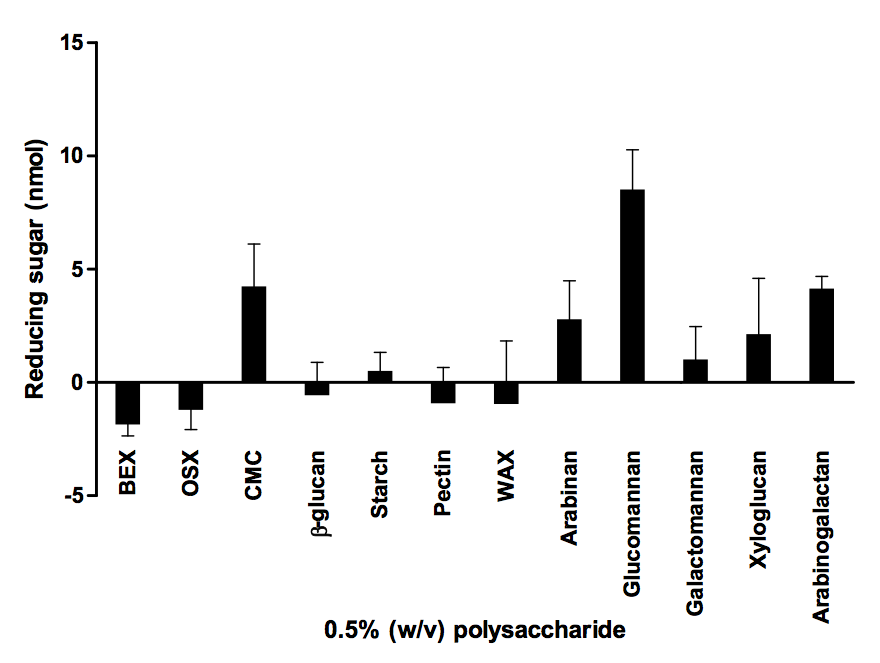

Supplement: Supplementary file 4 — Additional file 4: Fig. S4. FjoAcXE activity screen against 0.5% (w/v) of selected polysaccharides. Reactions (50 µL) contained 5 µg of FjoAcXE, 50 mM HEPES (pH 8.0), and 0.5% w/v of each substrate, and were incubated for 16 h at 30 °C. Reducing sugars were measured using 1% final PAHBAH reagent [58]. BEX = beechwood xylan (Sigma, X4252); OSX = oat spelt xylan (Sigma, X0627); CMC = carboxymethylcellulose (Megazyme, P-CMC4 M); β-glucan (low viscosity; from barley; Megazyme, P-BGBL); starch (from corn; Sigma-Aldrich, S4126); pectin (from apple; Sigma, 76282); WAX = wheat arabinoxylan (high viscosity; Megazyme, P-WAXYH); arabinan (from sugarbeet; Megazyme, P-ARAB); glucomannan (low viscosity; from konjac; Megazyme, P-GLCML); galactomannan (from guar, GD28; Megazyme, enzyme modified); xyloglucan (amyloid, from tamarind seed; Megazyme, P-XYGLN); arabinogalactan (acacia gum, Sigma, G9752). [file 13068_2018_1074_MOESM4_ESM.tiff]

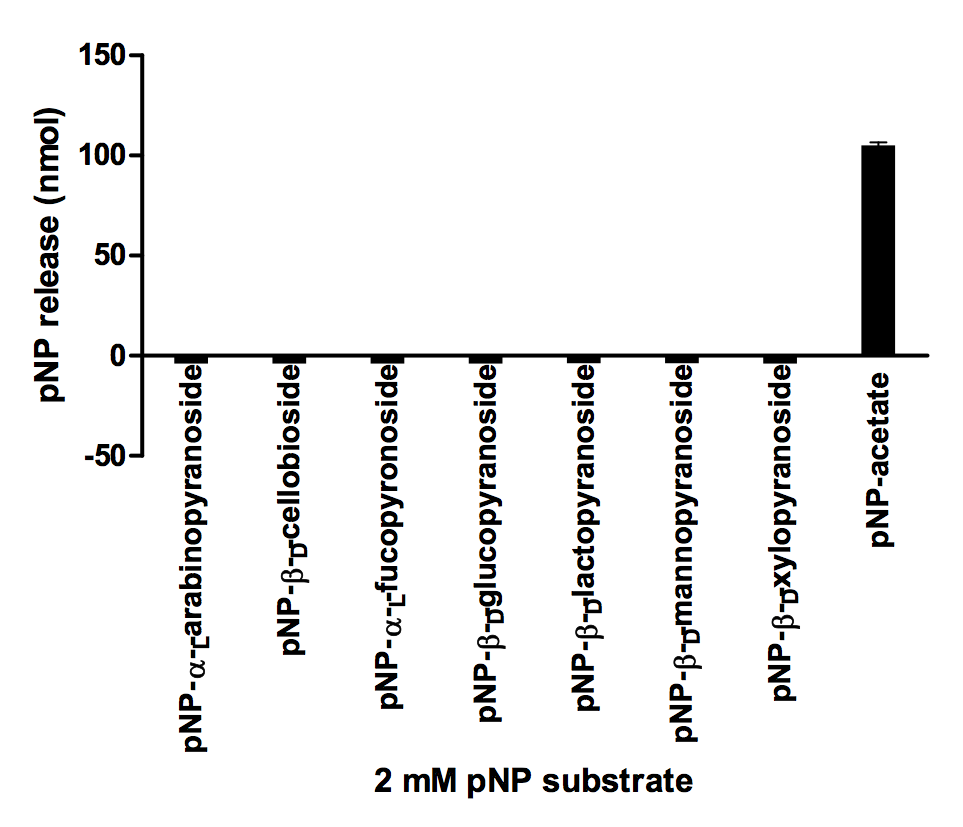

Supplement: Supplementary file 5 — Additional file 5: Fig. S5. FjoAcXE activity screen against selected pNP substrates. Reactions (200 µL) contained 5 µg of FjoAcXE, 50 mM HEPES (pH 8.0), and 2 mM of each substrate. Absorbance was measured after 2 h at 30 °C. [file 13068_2018_1074_MOESM5_ESM.tiff]

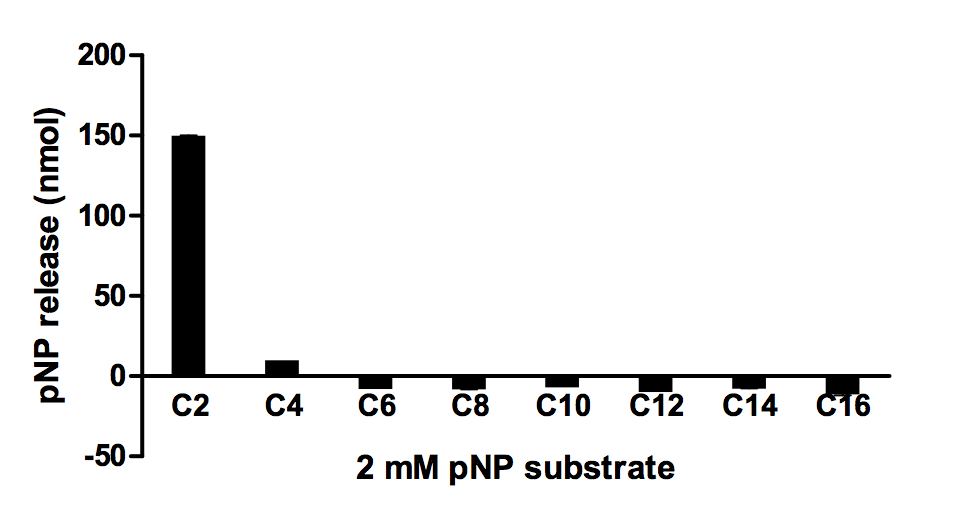

Supplement: Supplementary file 6 — Additional file 6: Fig. S6. Screen of FjoAcXEA activity towards selected pNP alkyl esters showing activity on short chain (< C4) substrates consistent with esterase rather than lipase activity. Reactions (200 µL) contained 0.5 µg of FjoAcXE, 50 mM HEPES (pH 8.0), and 2 mM of each substrate. Absorbance at 410 nm was measured after 2 h at 30 °C. pNP acetate (C2), pNP butyrate (C4), pNP hexanoate (C6), pNP octanoate (C8), pNP decanoate (C10), pNP dodecanoate (C12), pNP myristate (C14), and pNP palmitate (C16). n = 3; error bars correspond to standard deviation. [file 13068_2018_1074_MOESM6_ESM.tiff]
